# A decade of progress in marine evolutionary biology

**DOI:** 10.1111/eva.13523

**Published:** 2022-12-25

**Authors:** Pierre De Wit, Ellika Faust, Leon Green, Marlene Jahnke, Ricardo T. Pereyra, Marina Rafajlović

**Affiliations:** ^1^ Linnaeus Centre for Marine Evolutionary Biology University of Gothenburg Strömstad Sweden; ^2^ Department of Marine Sciences, Tjärnö Marine Laboratory University of Gothenburg Strömstad Sweden; ^3^ Department of Biological and Environmental Sciences University of Gothenburg Gothenburg Sweden; ^4^ Department of Marine Sciences University of Gothenburg Gothenburg Sweden

**Keywords:** CeMEB, evolutionary biology, marine evolution

## Abstract

This article summarizes the Evolutionary Applications Special Issue, “A decade of progress in Marine Evolutionary Biology.” The globally connected ocean, from its pelagic depths to its highly varied coastlines, inspired Charles Darwin to develop the theory of evolution during the voyage of the Beagle. As technology has developed, there has been a dramatic increase in our knowledge about life on our blue planet. This Special Issue, composed of 19 original papers and seven reviews, represents a small contribution to the larger picture of recent research in evolutionary biology, and how such advancements come about through the connection of researchers, their fields, and their knowledge. The first European network for marine evolutionary biology, the Linnaeus Centre for Marine Evolutionary Biology (CeMEB), was developed to study evolutionary processes in the marine environment under global change. Though hosted by the University of Gothenburg in Sweden, the network quickly grew to encompass researchers throughout Europe and beyond. Today, more than a decade after its foundation, CeMEB's focus on the evolutionary consequences of global change is more relevant than ever, and knowledge gained from marine evolution research is urgently needed in management and conservation. This Special Issue, organized and developed through the CeMEB network, contains contributions from all over the world and provides a snapshot of the current state of the field, thus forming an important basis for future research directions.

## 1 INTRODUCTION

This Special Issue is a celebration of the first European network for marine evolutionary biology, the Linnaeus Centre for Marine Evolutionary Biology (CeMEB), established in 2008 at the University of Gothenburg, Sweden. The creation of CeMEB was inspired by the societal need to better understand adaptation to changing marine environments and facilitated by funding from the Swedish Research Councils (see Johannesson et al., 2022; this issue, for more details). Key scientific topics addressed in the more than 500 CeMEB‐related publications to date include local adaptation, modeling of the distribution and genetic structure of marine species, and the incorporation of genomic information into conservation management.

The aim of this Special Issue is to gather contributions that advance the understanding of evolution in the marine realm and to bridge our knowledge gaps about processes such as adaptation to local environments, connectivity, intraspecific divergence, as well as the underlying ecological, physiological, and genetic mechanisms that influence these processes. Much of our empirical knowledge on evolutionary biology stems from terrestrial model systems, and yet a large part of the tree of life exists only in the marine environment (May, 1994). Consequently, it is critical to also develop new model systems from marine taxa. Marine Evolutionary Biology is a young field of research, historically considered a difficult‐to‐study special case of general evolutionary biology, yet it has expanded and matured rapidly in the past decades. This expansion, in part, stems from the development of novel sequencing technologies that allow for the study of less accessible systems, such as the marine realm, in more detail. This Special Issue provides a glimpse of the current state of Marine Evolutionary Biology research while delivering perspectives on the advances during the past decade that have led to the present state. More information on CeMEB is available at https://www.gu.se/en/cemeb‐marine‐evolutionary‐biology.

This Issue consists of 26 articles with perspectives, review articles, and original research in Marine Evolution. Perspective/review pieces include reflections on the development of Marine Evolutionary Biology research as a whole (Johannesson et al., 2022), as well as detailed considerations of marine evo‐devo research (Stracke & Hejnol, 2022), on divergence and speciation in the sea (De Jode et al., 2022), and on human‐mediated evolution (Touchard et al., 2022). Furthermore, several manuscripts describe how technological improvements have allowed for more in‐depth studies of genomic divergence (Pampoulie et al., 2022), the evolution of polymorphic traits (Gefaell et al., 2022), and the genomic mechanisms underlying phenotypic traits (Li & Hui, 2022). One major topic studied within the Marine Evolutionary Biology community is the interplay between genetic and plastic mechanisms in the processes of local adaptation, ecotype formation, and speciation, and how these processes may be impacted by, e.g., sexual selection, individuals' dispersal patterns, or species‐specific genomic properties, such as the size and distribution of chromosomal rearrangements (e.g., Faria et al., 2021; Ravinet et al., 2017). Herein, these processes are addressed in diverse empirical model systems ranging from diatoms to mollusks and fish (Green et al., 2022; Le Moan et al., 2022; Sefbom et al., 2022; Zhang et al., 2022), as well as in silico in two theoretical modeling studies (Eriksson et al., 2022; Marshall & Connallon, 2022). The link between genotype and phenotype is also the focus of several empirical studies here (Gefaell et al., 2022; Stenger et al., 2022; Walker et al., 2022), as is the seascape genomics of a wide range of taxa (Delaval, Bendall, et al., 2022; Delaval, Frost, et al., 2022; Fitz et al., 2022; Lapègue et al., 2022; Matias et al., 2022; Palumbi et al., 2022; Pampoulie et al., 2022), which has important implications for management of marine resources. Finally, the breadth of phylogenetic diversity and evolutionary history in the oceans is the topic of several articles focusing on the larval development of gastropods (Korshunova et al., 2022; Sun et al., 2022) and the evolution of small RNAs in cnidarians (Li & Hui, 2022). Although the work of this issue was initiated, coordinated, and edited by CeMEB, the contributions are from research groups throughout Europe, Asia, and North America. Eight of 26 contributions are authored by members of the CeMEB network.

## 2 OVERVIEW OF THE ISSUE

The field of Marine Evolutionary Biology has matured substantially since the founding of CeMEB in 2008. In order to gain a detailed historical perspective of this progress from the people involved, we consider it important to include the views of the founding researchers. The first article of the issue is therefore an invited perspective, which reflects on the networking and scientific activities of the Centre, co‐authored by the steering committee of the Centre from 2008–2018. Here, Johannesson et al. (2022) go from lessons learned to a look into the future and give their viewpoint on how the field of marine evolutionary biology has been, and still is, evolving. The authors state that the 10‐year funding of the Centre has allowed it to act like a “large sailing ship,” riding the winds of science forward, and highlighting the marine realm as an important focus for evolutionary biology. They discuss the importance of novel sequencing technologies and the difficulties of creating new marine model systems and reference genomes in species with complex genome arrangements, and conclude that while much has been learned since 2008, far more work is still needed to understand local adaptations to a fluctuating multivariate environment, the regulatory pathways behind genotype–phenotype interactions and, more broadly, the diversity of life in the oceans. These important topics are the main focus of this Special Issue.

### 2.1 Local adaptation to a multivariate environment

Genetic adaptation and phenotypic plasticity interact to regulate individuals' responses to environmental changes, determining how a population or species will evolve. In this Special Issue, responses of local populations to environmental drivers are addressed in several articles. Sefbom et al. (2022) address the paradox of marine organisms with high dispersal potential—in this case, the marine microalga *Skeletonema marinoi*—and nevertheless strong population structure and local adaptation. The authors used reciprocal transplants of multiple strains grown in different salinities and found that when grown alone, both marine and estuarine strains performed best in a high‐salinity environment. By contrast, when strains were allowed to compete, strains with marine genomic background performed better than estuarine strains in the marine environment. While this study highlights genetic mechanisms behind the local adaptation, other studies have pointed at epigenetic variation as another important mechanism for rapid acclimation to global change (Allendorf et al., 2022; Stajic & Jansen, 2021). Scheschonk et al. (2022) assessed the epigenetic methylome of the economically important kelp *Saccharina latissima* from two wild populations sampled at different latitudes (Germany 54° N; Svalbard 78° N) and tracked methylation/epigenetic changes in common‐garden cultivations at different temperatures. The latitudinal origin was associated with differences in the methylome and the results suggest that methylation mechanisms can also result in different and locally adapted “eco‐phenotypes.”

Common‐garden experiments are often used to evaluate the performance of individuals under future climate change. Walker et al. (2022) experimentally investigated bleaching resistance and recovery among colonies of the coral *Acropora hyacinthus* in Palau, by measuring coral bleaching, mortality, and skeletal growth. They showed that, although heat resistance and mortality were overall negatively correlated, the skeletal growth of less heat‐resistant corals was significantly faster than in corals highly resistant to heat stress. These findings suggest that heat‐stress capacity is costly, which may have a critical impact on future populations' resilience, arguing for the need to incorporate multiple resilience indicators in management actions.

Although common‐garden and reciprocal transplant experiments are powerful tools to test local adaptation and stress tolerance, the interpretation of results may be challenging. Plastic responses can be invaluable to mitigate species' vulnerability to rapid global change; however, Eriksson et al. (2022) argue that traditional approaches comparing reaction norms of organisms exposed to different environments may not account for the adaptive value of plastic responses. The authors supported this argument by comparing modeling simulations of adaptive vs fitness‐correlated traits. They concluded that some knowledge of the relationship between assessed traits and fitness is crucial to understand the adaptive nature of plasticity. The authors then applied the modeling insights in a reciprocal transplant experiment and inferred that *Idotea balthica* isopods from brackish water showed reduced adaptive plasticity compared with a marine population. Marshall and Connallon (2022) also propose a theoretical modeling approach to assess adaptation in species with complex life histories. Using an extension of Fisher's geometric model, the authors explored the correlations between traits and functions across different life stages to shed light on the evolutionary dynamics of these complex life histories. Their results showed that the emergence of fitness trade‐offs between stages may be common, either through divergent selection or mutation and, while evolutionary conflicts may intensify during adaptation, carry‐over effects can mitigate the conflict favoring survival in earlier life histories at the expense of later stages. Overall, this study implies that organisms with complex life histories could be more constrained in their capacity to adapt to global change than those with simple life histories.

### 2.2 Seascape approaches

The responses of populations to future climate change (in terms of adaptive capability, range shifts, and expansions of native and non‐native/invasive species) depend, in part, on individuals' dispersal abilities across the seascape (realized connectivity/gene flow). In this Special Issue, dispersal across the seascape and consequences for management are studied in multiple systems, including microalgae, corals, mollusks, and fish. Touchard et al. (2022) address marine evolution and local adaptation in ports and other areas, which are strongly impacted or rapidly changed by human activities. The authors discuss how rapid evolution in harbors, for instance, driven by adaptation to toxins or hybridizations, has been accelerated by a combination of human impacts, the mixing of native and invasive taxa, and elevated connectivity over large spatial distances due to boating activities. It is a fascinating discourse on what lessons marine evolutionary biology can learn from “biological portuarization,” the repeated evolution of marine species in port‐ecosystems and how researchers could best make use of ports by treating them as giant—and strongly replicated—mesocosm experiments for supporting predictive marine evolution. Parallel evolution is likewise one focal point of Lapègue et al.'s (2022) study on native flat oyster *Ostrea edulis* populations along the European coast. Here, a clear genetic break was found between the Atlantic and the Mediterranean, but oysters at the far ends of the distribution (North Sea—Black Sea) shared outlier loci exhibiting similar allele frequency shifts, indicating either a shared evolutionary history among the two areas, or parallel selective pressures at the range edges acting on standing genetic variation. Further, evolution at species range edges is empirically scrutinized in Green et al.'s (2022) study on the invasive round goby *Neogobius melanostomus*, which was tested for genomic and phenotypic differences across short spatial scales but in a strong environmental gradient. The findings showed both genotypic and phenotypic differences over surprisingly small scales. Multiple introductions together with strong selective sorting pressures on standing genetic variation were concluded to be a likely cause of this pattern in the gobies, just as in the oysters.

Physical separation of two populations inhabiting the marine environment can either occur abruptly through a geographic barrier as seen in the oysters above or gradually with increasing physical (or oceanographic) distance due to limitations in dispersal (classic isolation‐by‐distance). In two related articles in this Special Issue, Delaval, Bendall, et al. (2022) assessed dispersal and abundance in the blue skate, *Dipturus batis* in north‐east Atlantic waters, a species under continuous fishing‐ and by‐catch pressure. In one of the studies, they implemented a novel modeling approach using closely‐related individuals from fished samples, “Close‐kin mark‐recapture” (CKMR), to estimate demographic parameters relevant for conservation, e.g., adult breeding abundance and survival rates. Using this approach with SNP data from the blue skate samples, they found indications of stable population sizes across time series, while identifying site fidelity in the species, suggesting an area needed for the blue skate conservation (critical habitat). Traditional fisheries‐independent approaches evaluate several species at once and are often biased due to insufficient knowledge of individual species biology, while mark‐recapture methods are frequently insufficient given the usual low rates of tag and animal recapture. Thus, this modeling approach may represent a promising alternative for fisheries management. Taking a seascape genomic approach, Delaval, Frost, et al. (2022) also genotyped blue skates and correlated allele frequencies with environmental parameters. When characterizing contemporary population structure, the deep waters of Rockall Trough were identified as a barrier separating inshore (British Isles) and offshore (Rockall and Faroe) individuals, with small effective population sizes in some areas. The isolation of offshore populations compared with high coastal connectivity highlights the importance of bathymetric barriers, rather than isolation‐by‐distance, which has been observed in many other elasmobranchs. Fitz et al. (2022) focused on the population genetic patterns of the anemonefish *Amphiprion biaculeatus*, comparing different geographic determinants of genetic structure. Here, the authors present models detailing whether spatial distance or dispersal with currents best explained genetic separation in this rare reef fish and concluded that, while oceanographic currents best explained genetic patterns at large scale (>150 km), isolation‐by‐distance was a stronger determinant at smaller geographic scales.

In organisms with a mixture of long‐ and short‐range dispersal, population genetic patterns can become increasingly complex. Palumbi et al. (2022) present such a study case on south Pacific corals. Genetic differentiation in corals is usually found over long distances (hundreds or thousands of kilometers). However, the authors found mitochondrial genetic differentiation in the staghorn coral *Acropora hyacinthus* in Palau over much shorter distances (1–25 km), with closely‐related mitochondrial genomes more likely to co‐occur on the same reef than expected by chance. Additional data from distant colonies in American Samoa indicated higher differentiation between the Palau and American Samoa samples, but also some shared identical mitochondrial genomes. These findings were supportive of rare long‐distance dispersal in the studied coral populations and a stronger retention tendency of some genome sequences over others. Consequently, the authors call for monitoring the retention tendency and to use these data to aid assisted migration management plans. Matias et al. (2022) further illustrate the complexity of spatial genetic variation in corals. Using genome‐wide SNPs, the authors examined the genetic variation of the reef‐building coral *Acropora tenuis* and its associated endosymbiotic algae along the entire Great Barrier Reef. While this study found that the corals differentiated into three distinct genetic clusters associated with latitude and inshore–offshore reef position, the symbiont pool correlated with inshore–offshore environmental gradients. The environmental influence on the symbiont community composition supported the notion that the coral symbionts may contribute to coral adaptation to global change.

### 2.3 Ecotypes—Speciation

A central goal of evolutionary biology is to understand the formation of divergent populations and species and the drivers of this divergence. As tools and methods to address these questions have changed over time, De Jode et al. (2022) review a decade of marine divergence and speciation research combining genome‐wide data with demographic modeling to infer the demographic history of multiple marine species. Through this overview, they could show that while geographic barriers to gene flow do exist in the sea, divergence can also occur without strict isolation. Interestingly, most population pairs examined in this study exhibited heterogeneous gene flow, suggesting a predominance of semi‐permeable barriers during divergence. Another historical review of eco‐evolutionary divergence is outlined by Pampoulie et al. (2022), examining the past 60 years of management studies of Atlantic cod, *Gadus morhua*. Atlantic cod is heavily exploited across the northern Atlantic and with the advent of a fully assembled genome, genomic differences have been found between behavioral ecotypes that are either stationary or migratory. This study aims to provide insights into these ecotypes for more sustainable fisheries of the remaining stocks, while also reviewing the improvement of genomic methods over the time period.

The topic of advances in genomic methods is likewise explored in a study of the flat periwinkle *Littorina fabalis*, which displays a large and a dwarf ecotype occupying different microenvironments. Early genetic work found sharp allele frequency differences at the *arginine kinase* locus (*Ak*) along the environmental cline between these ecotypes (Tatarenkov & Johannesson, 1994, 1998). Using whole‐genome sequencing, Le Moan et al. (2022) revisited this historically studied system to increase our understanding of the *Ak* variation and its relationship to the divergence between the two ecotypes. Le Moan et al. (2022) not only found nine nonsynonymous substitutions, which were a perfect fit to the different migration patterns of the *Ak* alleles, but also discovered that the *Ak* alleles were located on different arrangements of a putative chromosomal inversion.

As most organisms depend on symbioses to function, divergence in host‐symbiont relationships can be just as important to fitness as within‐species genomic divergence among ecotypes. The genomic divergence between ecotypes in *Littorina* was found to be mirrored by their gut bacterial community composition by Panova et al. (2022). The authors found that the biofilm communities that *Littorina saxatilis* grazed on differed depending on habitat, which, in turn, explained gut community differences between snail ecotypes. This system now also offers the possibility to study the co‐evolution of gut microbiota and their hosts in the marine environment.

Sexual selection is one of the most important drivers of ecotype and sexual dimorphism formation, as well as a driver of speciation. In many species, males diverge into dominant and sneaker males that try to access spawning opportunities by disguising themselves as females (Khelifa, 2019). There is also substantial empirical evidence of sneaking males that have evolved larger testes and more sperm in response to higher sperm competition (Mank, 2022; Taborsky, 2008). However, it is less clear how such increased sperm competition may affect sperm performance, e.g., motility, longevity, and velocity. To investigate this, Kvarnemo et al. (2022) conducted a comparative study of the sand goby, *Pomatoschistus minutus*, where they found a clear difference in gene expression between testes of nest‐building and parasitic sneaker males. However, despite these differences, Kvarnemo et al. (2022) found no significant divergence between the two male morphs in sperm traits. The authors concluded that their results supported previous findings that natural selection is unlikely to result in the evolution of increased sperm performance in response to higher sperm competition. Comparing how closely‐related species respond to different environments is another insightful method to explore adaptive divergence. In this Special Issue, Zhang et al. (2022) investigated two sympatric sister species, *Crassostrea hongkongensis* and *Crassostrea ariakensis*, to unravel the phenotypic patterns and molecular mechanisms underlying salinity adaptation in marine mollusks. Physiological parameters such as growth rate and survival suggested higher fitness of *C. ariakensis* in high‐salinity and of *C. hongkongensis* in low‐salinity. Zhang et al. (2022) also found that the two species exhibited differential gene expression, with many differentially expressed genes involved in important salinity‐responsive pathways. These results may aid the assessment of the adaptive capacity of economically important marine invertebrates in the context of climate change.

### 2.4 Phylogenetic biodiversity of the oceans

Comparative developmental biology is another important aspect of evolutionary biology and Stracke and Hejnol (2022) argue that the inclusion of more marine taxa in developmental models is a crucial step in understanding animal evolution, as many phylogenetic lineages are only present in the oceans. They further discuss how technological advances in the past decade have expanded our knowledge of the diversity of developmental biology, allowing the inclusion of more marine taxa. As a study case of these advanced methods, Sun et al. (2022) used phalloidin staining, in‐situ hybridization, and confocal laser scanning microscopy techniques to study mesodermal development in embryonal *Lottia goshimai*, the second species of trochophore‐larval bearing gastropods to be studied to date. Also highlighting this need for alternative model taxa in evolution, Li and Hui (2022) provide an overview of small RNA (sRNA) biology in cnidarians, calling for more studies of sRNA in complementary eukaryotes. This is a timely piece that also shows the progress in sRNA studies as important forces in gene expression and genome stability of eukaryotes. In some cases, the combination of morphological and molecular data can show that taxonomic practices do not reflect evolutionary processes, a subject explored by Korshunova et al. (2022) in their review on the “lumpers & splitters” dilemma. Using the nudibranch mollusk genera *Catriona* and *Tenellia*, the authors demonstrate that fine‐scale taxonomic differentiation of traits is an important tool in the integration of morphological and molecular data, and how lumping diversity into a single taxon can lead to producing an oversized, unmanageable taxon of highly disparate species.

In the past, color patterning has been a trait often used in species delineation. However, color patterns may arise in diverse evolutionary ways in different taxa and are not necessarily always associated with speciation (e.g., Andrade et al., 2019). Gefaell et al. (2022) reviewed shell color polymorphism in marine gastropods, providing a summary of the patterns of color diversity in gastropod species, the underlying biochemical and genetic mechanisms in this trait, and the role of different evolutionary mechanisms in generating and preserving color polymorphism in gastropod taxa. Based on 61 studies, the authors conclude that natural selection is typically involved in the maintenance of shell color polymorphism in marine gastropods, although the roles of drift and gene flow were rarely considered or explicitly tested for in the reviewed articles. The diversity in color was also investigated by Stenger et al. (2022), who studied three shell color phenotypes of the pearl oyster *Pinctada margaritifera*. They used a pooled whole‐genome sequencing approach to investigate color‐associated SNPs in three wild populations and one hatchery. Their results not only confirmed known SNPs associated with pigment‐related genes but also identified new genes involved, as well as novel pathways. These findings are valuable for future breeding programs of pearl oysters.

## 3 DISCUSSION

Here we have gathered 26 articles that study different facets of evolution in marine environments. Increasing the knowledge on various aspects of evolution in the marine realm will be important to improve our understanding toward future responses to global change, expected outcomes of restoration programs, or appropriate management of economically important species. Broadly, the contributions to this issue can be grouped into four topics, each discussed below.

### 3.1 Experimental work involving common‐garden or reciprocal transplant experiments to study plasticity and local adaptation patterns

Marine evolutionary research is lagging behind its terrestrial counterpart, perhaps due to the difficulty of accurately simulating marine environments in laboratory conditions, or the complex life cycles (and sensitive larval stages) of many ecologically relevant species (Sanford & Kelly, 2011). The majority of marine studies on this topic have been limited in duration, number of drivers, and in the ability to extrapolate to fitness consequences over time. One crucial factor highlighted in this Special Issue is that the relationship between measured traits and fitness in common‐garden or transplant experiments needs to be well characterized to draw adequate conclusions about plastic or adaptive capabilities **(**Eriksson et al., 2022 **)**. The fitness value of a particular trait is often hard to determine and even more in an understudied ecological context. While the field is readily moving toward multi‐stressor experiments, entire life cycles, and ideally with realistic ecological frameworks (Riebesell & Gattuso, 2015), this is certainly not a trivial task. The next decade will prove determinant in large‐scale developments in this area.

### 3.2 Connectivity and seascape genomics

The decreasing costs of sequencing technologies have allowed this field to expand considerably during the past decade. Consequently, numerous studies are now available, which combine field‐collected samples and population genomic inferences with environmental data to examine both local adaptation and barriers to gene flow in the marine realm. Most analytical methods originally developed in terrestrial systems are also applicable to marine organisms, albeit considering that marine taxa may have large population sizes and rather different dispersal mechanisms compared with terrestrial species (Liggins et al., 2019). The complex life cycles of many organisms, the scarce knowledge about local temporal fluctuations in oceanic environmental parameters (especially in the coastal zone), and the lack of understanding of larval interactions with currents hamper the integration of genetic and biophysical connectivity studies (Riginos et al., 2016), although both approaches generally confirm each other (Jahnke & Jonsson, 2022; Legrand et al., 2022). Future collaborations between biologists and oceanographers will be crucial to solve these urgent issues, thereby providing managers and decision‐makers better tools to evaluate the effects of, e.g., marine spatial planning, restoration efforts, or climate change mitigation initiatives.

### 3.3 Ecotype formation and speciation

Despite the apparent lack of physical barriers in the oceans, studies have revealed a plethora of biological systems that include ecotypes with incipient barriers to gene flow. A classic example, first described already in 1990 (Johannesson & Johannesson, 1990) is the rough periwinkle, *Littorina saxatilis*, where nonrandom mating together with a highly heterogeneous environment has given rise to crab‐ and wave ecotypes. More recently, the ecotype concept has been applied to other organisms, as the contributions to this Special Issue have also been highlighted (De Jode et al., 2022; Pampoulie et al., 2022). For example, the Atlantic cod has been grouped into ecotypes based on behavioral differences (Knutsen et al., 2018). All these systems provide excellent opportunities for studying speciation in the marine environment, with genetic features playing important roles in forming reproductive barriers, such as chromosomal rearrangements (Faria et al., 2019; Johannesson et al., 2020; Matschiner et al., 2022). Another way to study speciation is through adaptive radiations such as the classic Darwin's finches (Grant & Grant, 2006) and cichlids in the African great lakes (Seehausen, 2004). In this issue, we highlight a marine example: the radiation of *Magallana* spp. oysters (formerly *Crassostrea*) along the Chinese coast (Zhang et al., 2022). Including marine examples in speciation genomics will clearly generate a more complete understanding of the process.

### 3.4 Phylogenetic biodiversity studies

The majority of all of the diversity of life exists in the oceans, exemplified by 80 % of all animal phyla only being solely marine (May, 1994). Only a very small part of this diversity has been studied and even less has been characterized at the genomic level. Indeed, the number of marine species with reference genomes available has increased dramatically over the past decade, including through efforts by members of the CeMEB community. Researchers from CeMEB sequenced the genomes of eight ecologically significant species, all present along the environmental gradient stretching into the “Darwinian laboratory” of the Baltic Sea (Johannesson et al., 2020). The chosen species were seen as ecologically and evolutionary relevant for different aspects such as their phylogenetic diversity (*Pomatoschistus minutus*; Leder et al., 2021, *Littorina saxatilis*; Westram et al., 2018), stress tolerance (*Balanus improvisus*; Sundell et al., 2019), coastal dynamics (*Fucus vesiculosus*; Kinnby et al., 2020, *Idotea balthica*; De Wit et al., 2020), development (*Amphiura filiformis*; Dupont & Thorndyke, 2006) and even as tractable model organisms for genetic modifications (*Skeletonema marinoi*; Johansson et al., 2019). The more recent Earth BioGenome project initiative (https://www.earthbiogenome.org/) aims to sequence genomes of all eukaryotes and has certainly contributed to the task in the marine realm. Nevertheless, much painstaking work is still needed to link these new genome sequences to functional aspects of the organisms' physiology. As gene function may differ across taxa (Jax et al., 2018), newly sequenced organisms must be experimentally tested for this using different gene modification approaches in combination with developmental biology work (Santos et al., 2015), as well as novel developments in microscopic imaging techniques (Stracke & Hejnol, 2022). To accomplish these crucial but still rare types of studies, stable cultures of new model organisms from distant branches on the tree of life will be much needed.

## 4 CONCLUSION

The field of marine evolutionary research has seen huge progress both in basic and applied science over the last decade, contributed by the increasing studies of nonmodel organisms, as well as technological and analytical developments. This Special Issue highlights that the current focus of marine evolutionary research is incredibly broad and highly relevant to the societal challenge of how biodiversity will reshape and transform under the global change.

## CONFLICT OF INTEREST

The authors declare no conflicts of interest.

## Data Availability

Data sharing is not applicable to this article as no new data were created or analyzed in this study.
